# Ethnobotany, Ethnopharmacology, and Phytochemistry of Medicinal Plants Used for Treating Human Diarrheal Cases in Rwanda: A Review

**DOI:** 10.3390/antibiotics10101231

**Published:** 2021-10-09

**Authors:** Noel Gahamanyi, Emmanuel Munyaneza, Emmanuel Dukuzimana, Naasson Tuyiringire, Cheol-Ho Pan, Erick V. G. Komba

**Affiliations:** 1 SACIDS Foundation for One Health, College of Veterinary Medicine and Biomedical Sciences, Sokoine University of Agriculture, Chuo Kikuu, Morogoro P.O. Box 3015, Tanzania; ekomba@sua.ac.tz; 2 Natural Product Informatics Research Center, KIST Gangneung Institute of Natural Products, Gangneung 25451, Korea; 3 Rwanda Cultural Heritage Academy/Museum of Environment, Kigali P.O. Box 6397, Rwanda; munyaneza4@gmail.com; 4 School of Medicine and Pharmacy, College of Medicine and Health Sciences, University of Rwanda, Kigali P.O. Box 3286, Rwanda; emmylavie3@gmail.com; 5 School of Nursing and Midwifery, College of Medicine and Health Sciences, University of Rwanda, University Avenue, Butare P.O. Box 56, Rwanda; ttijonason@gmail.com

**Keywords:** diarrhea, dysentery, traditional medicine, socio-culture, phytochemicals, Rwanda

## Abstract

Diarrhea, often caused by microorganisms, has been associated with high morbidity and mortality in Africa. Increased rates of antimicrobial-resistant pathogens have reignited the quest for alternative therapies. This review aimed at identifying medicinal plants used in the treatment of human diarrheal cases in Rwanda and analyzing their ethnobotany, ethnopharmacology, and phytochemistry. We searched PubMed/Medline, Google Scholar, ScienceDirect, and the Web of Science for published articles on medicinal plants used to treat diarrhea in Rwanda. Additionally, specialized herbarium documents of different institutes were reviewed. Articles were assessed for relevance, quality, and taxonomical accuracy before being included in this review. Overall, 63 species of medicinal plants belonging to 35 families were recorded. Asteraceae was the predominant family with six species, followed by Fabaceae and Lamiaceae, with five species each. The most reported species with anti-diarrheal properties were *Vernonia amygdalina* Delile, *Tetradenia riparia* (Hochst.) Codd, *Clerodendrum myricoides* R. Br. and *Chenopodium ugandae* (Aellen) Aellen. Leaves (66.7%) and roots (17.5%) were the commonly used plant parts in the preparation of medicine. Phytochemicals from medicinal plants with antidiarrheic activities were sesquiterpene lactones (*V. amygdalina*); terpene, sterols, saponosides, and flavonoids (*C. ugandae*); saponins and tannins (*T. riparia);* and tannins, flavonoids, and alkaloids (C. *myricoides)*. Six studies tested the antimicrobial activities of the plants against bacteria and viruses known to cause diarrhea. *Erythrina abyssinica*, *Euphorbia tirucalli*, *Dracaena afromontana*, and *Ficus thonningii* are socio-culturally important. Further research on toxicity and posology is needed to ensure the safety of medicinal plants.

## 1. Introduction

Diarrheal diseases are among the major etiologies of morbidity and mortality worldwide, with most of the cases occurring in low- and middle-income countries (LMICs), particularly in Sub-Saharan Africa (SSA) and South Asia [1,2,3]. Although diarrhea affects people of all ages, it leads to complications in children under five years of age, including malnutrition, growth retardation, and reduced cognitive development [4,5,6]. Diarrhea, often defined as “the passing per day of three or more loose or watery stools” [7,8,9], is associated with various risk factors, including unsafe water, poor sanitation, and childhood wasting, which are common in LMICs [3]. The three major forms of diarrhea include (i) the acute watery diarrhea characterized by dehydration; (ii) persistent diarrhea lasting for more than two weeks and associated with poor absorption, loss of nutrients, and wasting; and (iii) bloody diarrhea caused by poisons, pathogens, drugs, or acute inflammations, leading to intestinal damage [4]. In Rwanda, despite efforts in preventing childhood diarrheal deaths through rotavirus immunization and making health sector reforms, diarrhea ranks as the third cause of childhood morbidity and mortality [10,11].

Several enteric pathogens (bacteria, viruses, and protozoa) have been associated with diarrhea via the ingestion of contaminated food [12]. The commonly reported bacteria include different strains of *Escherichia coli*, *Shigella* spp., *Vibrio cholerae*, *Campylobacter* spp., *Salmonella* spp. and *Yersinia* spp. [12]. The major implicated viruses are rotavirus, adenovirus, and hepatitis A virus [12,13], while parasites include *Entamoeba histolytica* and *Giardia* spp. [13,14].

Undiagnosed cases of diarrhea are treated with different antimicrobials, contributing to the accelerated rates of bacteria that are resistant to a few existing antimicrobials [15,16]. For instance, increased resistance to ciprofloxacin has been partly attributed to its overuse in treating diarrhea of unknown etiologies [16]. Other contributors to antimicrobial resistance include natural mutations in pathogens, misuse of antimicrobials in animal husbandry, and the use of counterfeit drugs [15,17]. Drug discovery is very slow compared to the exponential increase in the number of antimicrobial-resistant (AMR) pathogens [15]. Therefore, with the ending era of conventional antimicrobials [18], there is a pressing demand to develop new antimicrobial agents if we are to cope with the threat of multidrug-resistant (MDR) pathogens [14,19].

Since ancient times, medicinal plants have been known as raw materials for the manufacturing of over one-third of currently used antimicrobial agents, and modern allopathic medicine has its roots in traditional medicine [15,20,21]. The literature shows that over 80% of currently used drugs are from natural products (herbal medicines, microbes, or their bioactive compounds) [9]. Despite a rich plant diversity (45,000 species) in Africa, data on their medicinal use is only available for 5000 species [22]. As of recently, a few anti-diarrheal drugs from plants include aesculetin from *Frazinus rhychophylla*, emetine from *Cephaelis ipecacuanha*, agrimophol from *Agrimonia supatoria*, berberine from *Berberis vulgaris*, hemsleyadin from *Hemsleya amabilis*, and neoandrographolide from *Andrographis paniculata* [23].

In SSA, rural households depend on plant sources for food, animal feeds, and medicines [24]. Complementary and alternative medicine (CAM) is defined as “a group of diverse medical and healthcare systems, practices, and products that are not generally considered part of conventional medicine” [25]. The estimate of people relying on CAM for basic health services worldwide and in Africa is 80% and 88%, respectively [26,27,28]. In East Africa, the population using traditional medicine, especially in rural areas, is over 80% [29]. The preference for folk medicine over conventional health services has been motivated by its easy accessibility, low cost, social-cultural influence, and the belief that herbal medicine is regarded as safe [2,29]. However, CAM faces various challenges related to its limited understanding by patients and low acceptance by conventional healthcare providers [30]. The use of CAM may lead to a drop in using conventional healthcare systems, which may increase morbidity and mortality due to complications that cannot be handled by traditional healers.

Since 2000, the World Health Organization (WHO) recommended that LMICs integrate CAM into their national health systems but only a limited number of countries (Tanzania, South Africa, Sierra Leone, Cameroon, Ethiopia, and Ghana) have managed to do so [30]. Africa is considered a cradle of several medicinal plants and a source of cultural diversity [31]. The integration of CAM into national health systems would help to prevent its complete disappearance as a result of being orally transferred from parents to their offspring [30,32,33]. In Rwanda, traditional medicine has been exercised by traditional healers known as *abavuzi gakondo* to treat various diseases of both humans and livestock [33,34]. However, research on traditional medicine has dated back to 1972 and it has helped to transform its image from a magical art to a trusted science [35]; it is estimated that 70% of the population in Rwanda uses traditional medicine [36]. The over-exploitation of medicinal plants for commercial purposes and the accelerated level of urbanization threaten the extinction of this precious heritage [37,38]. The European colonization introduced several invasive species which led to a significant decline in indigenous plants up to the verge of extinction [38]. The use of medicinal plants was attributed to poor people and was regarded as witchcraft [34]. In Rwanda, CAM was run through the University Research Centre for Pharmacopoeia Knowledge and Traditional Medicine (CURPHAMETRA), which was under the Institute of Scientific and Technological Research [39]. Despite not being well-integrated into the Rwandan healthcare system, a national policy of traditional, complementary, and alternative medicine has been established [40].

In Africa, the culture of collecting and using medicinal plants has been transferred orally throughout generations [37]. Globally, there is a shortage of information on traditional medicine available for research purposes [36]. The association of systematics, vernacular names, and indigenous uses of medicinal plants should be seen as paramount to either protect traditional knowledge or lay a foundation for further studies [29]. It is reported that successful research in ethnopharmacology requires the concerted efforts of botanists, chemists, taxonomists, pharmacologists, tradipractitioners, and beneficiaries [37]. Traditional medicine still faces challenges, including the scarcity of medicinal plants, the absence of harmonized preparation methods and quality control measures, and poor storage conditions [23].

The Diarrheal Disease Control Programme of the WHO advocates the use of traditional folklore medicines in the control and management of diarrhea [7,23]. Traditional healers who were treating diarrhea were coincidently curing dysentery, which is an acute diarrheal illness [7,37]. It is also reported that the line of distinction between diarrhea and dysentery is minor, which would justify the use of the same plants in treating both ailments [37]. In Rwanda, several medicinal plants have been used to treat diarrhea, but research on their chemical profiles and posology is limited [41]. To avoid risking a permanent loss of this rich and orally inherited tradition, there is a pressing demand to record this information [32]. With this background, this review aimed at identifying medicinal plants used in the treatment of human diarrheal cases in Rwanda and analyzing their ethnobotany, ethnopharmacology, and phytochemistry. This review may serve as a baseline for further research to discover and develop new drugs to treat various diseases, with diarrheal cases in particular.

## 2. Results

### 2.1. Classification of Medicinal Plants Used to Treat Diarrheal Cases in Rwanda

The present study identified 63 medicinal plant species belonging to 35 families as being traditionally used in Rwanda to treat diarrheal cases. Of the 63 species, 45 (71.4%) and 9 (14.3%) were reported to treat diarrhea and dysentery, respectively. Six (9.5%) medicinal plants are used to treat diarrhea and dysentery while three (4.8%) medicinal plants are used to treat cholera. The Asteraceae was the predominant family with six species, followed by Fabaceae and Lamiaceae, with five species each. The species most reported as having anti-diarrheal properties were *Vernonia amygdalina* Delile (umubirizi), *Tetradenia riparia* (Hochst.) Codd (umuravumba)*, Clerodendrum myricoides* R. Br. (umukuzanyana), *Tithonia diversifolia* (ikicamahirwe), *Cyathula uncinulata* (Schrad.) Schinz (igifashi), *Chenopodium ugandae* (Aellen) Aellen (umugombe), *Clematis hirsuta* Guill. & Perr. (umunkamba), and *Leucas martinicensis* (Jacq.) R. Br. (akanyamapfundo) (Table 1). The identified plants may be channeled toward research in drug discovery, and knowledge of their phytochemicals would be of paramount importance, which may attract pharmaceutical companies’ interests.

### 2.2. Preparation and Posology of Some Medicinal Plants Used for Treating Diarrheal Cases

Leaves were the most used parts of the plants (66.7%), followed by roots (17.5%). Other parts of the plants, including the bark of the stem, bark root, and whole plants, were also reported to be used in some cases. The preparation and posology of some medicinal plants used in treating diarrhea are depicted in Table 2. The dose and frequency of taking the solutions made from medicinal plants were only reported in two articles, referring to a cup in the morning and another one in the evening, or spoons of the medicine taken three times per day orally [43,49]. However, there is no standardization of the dose and frequency of administration. Some of the extracts were taken in combination with other plants as a concoction.

### 2.3. Assessment of the Antimicrobial Activity of Medicinal Plants Used in the Treatment of Human Diarrheal Cases in Rwanda

Six articles tested the antimicrobial activities of medicinal plants against diarrhea-causing agents. Twenty (20) medicinal plants were tested for antibacterial activity against *Shigella* and *Salmonella* species and anti-diarrheal activity in mice. Of the 20 medicinal plants, 65% exhibited anti-diarrheal activities, with *Myrica kandtiana* inhibiting the growth of several bacteria [44]. However, another study reported that all seven medicinal plants tested were not effective against *E. coli* and *Salmonella* [48]. Furthermore, different studies reported the anti-diarrheal activities of *Bidens pilosa*, *Tetradenia riparia* [46], and *Chenopodium ugandae* [56]. Mugiraneza et al. [56] reported the in vitro activity of *Chenopodium ugandae* and *Erythrina abyssinica* against *Salmonella* and *Shigella* species. Other medicinal plants reported to have antimicrobial activities were *Acacia sieberiana, Vernonia amygdalina,* and *Vernonia aemulans* (antiviral); *Clerodendrum myricoides* and *Euphorbia hirta* (antibacterial and antiviral); *Hibiscus surattensis*, *Tetradenia riparia*, and *Thunbergia alata* (antibacterial). Cos et al. [51] reported the antiviral and antibacterial activities of *Tithonia diversifolia* and *Chenopodium ugandae*. Strong antifungal activity was reported for *Clematis hirsuta* against *Candida albicans* [51].

### 2.4. Ethnopharmacology and Phytochemistry of Anti-Diarrheal Plants in Rwanda

Drug manufacturing using medicinal plants requires ethnopharmacological and phytochemistry studies that help in the validation process [57]. One of the extensively studied plants is *Tetradenia riparia*, for which some compounds (umuravumbolide and deacetylumuravumbolide) are named using root words of the Kinyarwanda language, and it is available in home gardens [58]. Recently, the anti-helminthic activity of *T. riparia* was found to be associated with 8(14),15-sandaracopimaradiene-7α,18-diol [59]. Furthermore, saponins and tannins from *Tetradenia riparia* have anti-helminthic activity [45,59].

The antibacterial activity of *Vernonia amygdalina* has been associated with sesquiterpene lactones (vernodalinol, vernolepin, vernomygdin, hydroxyvernolide, vernolide, and vernodalol) [60]. *Euphorbia hirta* and *Psidium guajava* are rich in quercitrin, which was found to stop diarrhea in mice, while *Mangifera indica* showed a high content of tannins, associated with astringent and anti-inflammatory effects [61].

*Tetradenia riparia*, *Leucas martinicensis*, and *Monechma subsessile* help to relieve gastrointestinal pain and combat diarrhea. All of these plants were found to have an antispasmodic activity [37,41]. *Chenopodium ugandae* is known to be rich in terpene, sterols, saponosides, flavonoids, and traces of alkaloids while flowers of *Erythrina abyssinica* are rich in saponosides [56].

*Clerodendrum myricoides* has been reported to exhibit strong antibacterial activities against both Gram-positive and Gram-negative bacteria, which are attributed to high amounts of tannins, flavonoids, and alkaloids, with traces of terpenoids and saponins [62].

### 2.5. The Role of Medicinal Plant Species in Social, Ecological, and Cultural Welfare

One study reported that some medicinal plants are also used for cultural and social purposes. For instance, *Ficus thonningii* Blume (umuvumu) is used in the fermentation of banana wine, and it was planted at the main gate as a symbol of protection and respect to family belongings. *Erythrina abyssinica* Lam. ex Dc. (umuko) is planted in the compound of the house for social and spiritual protection against devils. *Erythrina abyssinica* and *Ficus thonningii* are important for rituals and social ceremonies [33].

*Prinus africana* was reported to be a threatened species [38], but was later reported as extinct in Buhanga sacred forest due to the over-exploitation of both its leaves and bark as ingredients, while *Chenopodium ugandae* is on the list of priority species, for which the preservation of stored grains would prevent them from going extinct [33]. 

The populations of *Euphorbia tirucalli*, *Erythrina abyssinica*, *Ficus thonningii*, and *Dracaena afromontana* have heavily declined due to a shift from fencing with plants species to brick and concrete-walled fences [38].

## 3. Discussion

The current review discusses the ethnobotany, ethnopharmacology, and phytochemistry of medicinal plants that are commonly used in treating diarrheal cases in Rwanda. The results showed that the most reported species with anti-diarrheal properties include *Vernonia amygdalina, Clerodendrum myricoides*, *Chenopodium ugandae,* and *Chenopodium ambrosioides* L. These species have been reported in East Africa [62,63,64], in other parts of Africa [65], and in Mexico as well as Brazil [66,67] for treating diarrhea. *Psidium guajava* L. is known for the treatment of diarrhea in Burundi [67], Tanzania [63], and in various tropical countries [61,67]. 

Some medicinal plants, including *Mitragyna rubrostipulata* Havil, *Tragia brevipes* Pax, *Euphorbia hirta*, *Chenopodium ugandae*, and *Psidium guajava*, are used to treat diarrhea, dysentery, cholera, or gastroenteritis in general [33,42,44]. This reflects slight differences among various forms of diarrhea or the limited knowledge of these forms by traditional healers [34,68]. *Psidium guajava* has previously been reported to be used in the treatment of dysentery, gastroenteritis, stomach aches, and indigestion [9]. This may be explained by the fact that the same plant may contain various phytochemicals which are active against different diseases [68]. However, it is also possible that a single phytochemical may be effective against several diseases.

Most of the reviewed articles were published before 2005, showing gaps to be filled by research. In Rwanda, there is a lack of university programs specific to traditional medicine that would attract youth to research this re-emerging field of study. We hope that the national policy on traditional, complementary, and alternative medicine established in 2019 [40] will be followed by further steps towards the full integration of CAM into existing healthcare systems. In Africa, only 22 member states had included traditional medicine in the curricula of university health sciences by 2018 [69]. Some of the challenges highlighted by African governments in integrating CAM into existing healthcare systems were a lack of research data, financial support, education, and training for CAM providers, and a lack of appropriate mechanisms to control and regulate herbal products [69]. Despite its rich plant biodiversity [22], Africa is far behind Europe and Asia concerning natural products that are commercialized or traded [31].

The preparation of medicine from plants starts with the collection of different raw materials, such as plant roots, leaves, and bark [36]. In this review, it is reported that leaves (66.7%) and roots (17.5%) were the most used parts for the preparation of medicine, which concurs with previous reports from Tanzania [63], Uganda [29], Kenya [64], South Africa [70], and Ethiopia [36]. The use of leaves over roots and whole plants is a sustainable way of preserving some rare medicinal plants prone to extinction, as it allows for the long-term survival of medicinal plants [71]. The use of leaves is less damaging when compared to the use of roots and bark, which negatively affects the conservation of medicinal plants [64]. However, roots and bark are sometimes preferred by traditional healers due to their easy storage and transport when compared to leaves [68]. Therefore, the training of traditional healers and other people involved in the harvesting medicinal plants in methods that are less damaging to plants and suitable for long-term harvesting is important [23]. 

The predominant families of medicinal plants used in the treatment of diarrhea in Rwanda were Asteraceae, Fabaceae, Lamiaceae, and Euphorbiaceae, which concur with previous studies in Uganda [29], Ethiopia [36], and Brazil [57]. The Asteraceae family is the second largest family of Angiospermae with a worldwide distribution, and it is well-known for various phytochemical properties [72]. It is known that plant species contribute differently to an ecosystem and that the dominant ones may shape the community structure and diversity based on their high biomass, high productivity, and other traits [73].

The preparation and administration of plant extracts for the treatment of diarrhea vary with plant families and species. However, decoction was the preferred preparation method, while the oral route was the most reported administration route. It is known that boiling allows for the extraction and preservation of herbal medicines for a longer time when compared to cold extraction [29]. The addition of water, salt, milk, or banana highlights the importance of restoring minerals, water, and other nutrients lost due to diarrhea, and improve the bitter taste of some extracts [29,40,61,64]. Some extracts are used in a mixture, which makes it difficult to know the contribution of each to the treatment [46]. Therefore, the phytochemicals of each medicinal plant need to be isolated and chemically characterized.

Although medicinal plants are used in treating diarrhea, a limited number of them have undergone validation through clinical trials, highlighting the risk of using them [9]. The knowledge of using medicinal plants has been transferred from generation to generation through verbal communication [30,32]. There is a possibility of the original formula having been altered due to the lack of a written document. Additionally, the limited number of studies on the toxicity of medicinal plants underlines the steps to be covered before the integration of traditional medicine into conventional medicine [74]. For instance, some medicinal plants may possess mutagenic and carcinogenic compounds, which may show their effects over long periods [65]. Lastly, preparation methods are not standardized, which may influence the effectiveness of the medicinal extracts [23,36]. Therefore, the use of medicinal plants is important in healthcare systems, but their negative effects need to be well-known and addressed. Short-term and long-term health complications may arise from the improper use of medicinal plants, putting the lives of patient at risk [36].

It is important to check the antimicrobial activities of plants known to treat diarrhea using diarrhea-causing pathogens. This review found that two studies did not report any activity against the Gram-negative bacteria used [48,51]. It is known that the source of plants and extraction methods may affect the screening results [75]. The use of agar-based methods instead of broth microdilution in screening for antimicrobial activities is not suitable, as some bioactive compounds are non-polar and do not diffuse well in agar media [19,76]. Phytochemical characterization studies are limited in Rwanda, which is somewhat problematic as they are crucial to the discovery of new drugs from medicinal plants [34]. This study highlighted the studied phytochemicals with anti-diarrhea activities. Flavonoids are reported to have anti-diarrheal activity by promoting water and ion reabsorption in the colon [61], while 8(14),15-sandaracopimaradiene-7α,18-diol proved to be anti-helminthic [59]. This shows that new drugs can be made from those plants which would help in curbing the threat of antimicrobial resistance. However, further investigations need to be carried out to elucidate the in vivo efficacy, safety, mechanisms of action, and clinical trials [74,77]. This would pave the way for the integration of traditional medicine into national healthcare systems and reduce the high price of existing antimicrobials. 

Efforts to preserve endangered species such as *Prinus africana,* *Euphorbia tirucalli*, *Erythrina abyssinica*, *Ficus thonningii*, and *Dracaena afromontana* are important based on their cultural, social, ecological, and medicinal values [8,38,78]. Converting wild habitats, such as forests, into agricultural lands and for commercial purposes are among the major contributors to the steep decline in medicinal plants and trees [31,77]. Domestication and cultural integration of some medicinal plants would prevent their extinction [33,35]. Conservation programs should also be given priority as these medicinal plants are associated with the culture and history of Africa, and Rwanda in particular [79]. Pharmaceutical companies are advised to have medicinal plant gardens and to be involved in training both traditional healers and their dealers on suitable harvesting methods and safety issues along the whole production chain [23].

## 4. Materials and Methods

The information about medicinal plants used for treating diarrheal cases was gathered through the search of articles published either in English or French in PubMed using the following search strategy: (((Medicinal [All Fields] AND (“plants” [MeSH Terms] OR “plants” [All Fields])) AND (“Rwanda” [MeSH Terms] OR “Rwanda” [All Fields])) AND (“diarrhoea” [All Fields] OR “diarrhea” [MeSH Terms] OR “diarrhea” [All Fields])) AND (“diarrhoea” [All Fields] OR “diarrhea” [MeSH Terms] OR “diarrhea” [All Fields]). Then, edited search terms were used to obtain more articles from Google Scholar, Web of Science, and ScienceDirect. Initially, 1005 articles (Google Scholar (856), PubMed (73), ScienceDirect (64), and Web of Science (12)) were obtained and the lists of references were screened to get eight (8) additional articles. All articles were screened, and only 17 articles discussing the ethnobotany, ethnopharmacology, and phytochemistry of medicinal plants used to treat diarrhea in Rwanda were considered in this review after removing duplications and irrelevant articles (discussing other disease conditions or not related to Rwanda). The review articles involved were published between 1970 and 2018.

## 5. Conclusions

The current review discusses the medicinal plants used for treating human diarrheal cases in Rwanda, and the commonly used medicinal plants are *Vernonia amygdalina* Del., *Clerodendrum myricoides* R. Br., *Chenopodium ugandae* (Aellen) Aellen, and *C. ambrosioides*. There is a limited number of medicinal plants for which antimicrobial susceptibility profiling and phytochemical characterization have been carried out. Leaves and roots are the most used parts, highlighting the need for sustainable harvesting. Screening all the medicinal plants used in treating diarrhea would provide a complete list of essential plants that can be explored by pharmaceutical companies. Anthropogenic activities contribute to a decline in the populations of some medicinal plant species (*Prinus africana,* *Euphorbia tirucalli*, *Erythrina abyssinica*, *Ficus thonningii*, and *Dracaena afromontana*) which are of socio-cultural value. Further studies on the phytochemical characterization, in vivo efficacy, safety, the mechanisms of action, and clinical trials are encouraged. Lastly, conservation efforts are needed to preserve the species whose populations are considerably declining.

## Figures and Tables

**Table 1:** Medicinal plants used to treat diarrheal cases in Rwanda.

Family	Species	Vernacular Name	Use	Used Part	Reference
Asteraceae (6)	*Guizotia scabra* (Vis.) Chiov.	Igishikashike	D	L	[42]
	*Senecio cydoniifolius* O. Hoffm. Ex Engl.	Irarire	D	L	[43]
	*Tithonia diversifolia* (Hemsl.) A. Gray	Ikicamahirwe/kimbazi	D	L	[44]
	*Vernonia aemulans* Vatke	Idoma	D	L	[42,43]
	*Vernonia amygdalina* Delile	Umubirizi	D	L and F	[42,43,44,45]
	*Vernonia pogosperma* Klatt	Umubimbafuro	D	L	[42,45]
Lamiaceae (5)	*Clerodendrum myricoides* R. Br.	Umukuzanyana	D	R, BS	[42,44,46,47,48]
	*Leucas martinicensis* (Jacq.) R. Br.	Akanyamapfundo	DDC	F	[44,46]
	*Plectranthus laxiflorus*	Umukuzanyana	D	L	[49]
	*Tetradenia riparia* (Hochst.) Codd	Umuravumba	D	R, BR, and L	[46,47,50]
	*Thymus vulgaris* L.	Timu	D	ND	[35]
Fabaceae (5)	*Crotalaria mesopontica* Taub.	Akayogera	D	L	[46]
	*Entada abyssinica* A. Rich	Umusange	D	R, S, and L	[42]
	*Erythrina abyssinica* Lam. ex DC.	Umuko	Dysentery	L	[46]
	*Indigofera asparagoides* Taub	Kizibana	D	H	[42]
	*Senna septemtrionalis (Viv.) H.S.Irwin & Barneby*	Umukubanzoka	D	L	[33]
Euphorbiaceae (4)	*Euphorbia hirta* L.	Nyaantuku	DD	WP	[42]
	*Euphorbia tirucalli* L.	Umuyenzi	D	L	[42]
	*Phyllunthus bequaertii* Rob.	Uruheza	DC	S	[44]
	*Tragia brevipes* Pax	Isusa	DD	L	[33,44,46]
Amaranthaceae (3)	*Chenopodium procerum* Hoehst. ex Moq./*C. ambrosioides* L.	Umwisheke	DD	L	[33,44,46]
	*Chenopodium ugandae* (Aellen) Aellen	Umugombe	DD	L	[33,44,46,51]
	*Cyathula uncinulata* (Schrad.) Schinz	Igifashi	D	L	[46,51]
Malvaceae (3)	*Hibiscus fuscus* Garcke	Umutozo	D	L	[46,51]
	*Hibiscus surattensis* L.	Urubebwa	D	L	[46]
	*Triumfetta rhomboidea Jacq.*	Umushyigura	D	PRW	[46]
Acanthaceae (2)	*Monechma subsessile* (Olive) C.B. Clarke	Umubazi	D	L	[50]
	*Thunbergia alata* Bojer ex Sims	Nkurimwonga	D	L	[46]
Polygonaceae (2)	*Rumex abyssinicus Jacq.*	Igifumba	Dysentery	R	[46]
	*Rumex usambarensis (Engl.) Dammer*	Umufumbageshi	D	L	[46]
Myrtaceae (2)	*Psidium guajava* L.	Ipera	Dysentery	L	[44]
	*Syzygium parvifolium* (Engl.) Mildbr.	Umugote	D	B	[35]
Anacardiaceae (2)	*Rhus vulgaris* Meikle	Umusagara	D	L	[49]
	*Mangifera indica* L.	Umwembe	D	L	[43]
Rubiaceae (2)	*Rubia cordifolia* L.	Umukararambwe	D	R	[46]
	*Mitragyna rubrostipulata* Havil	Umuziwaziwa/umuzibaziba	DD	R, B, and L	[42,46]
Mimosaceae (2)	*Acacia sieberiana* DC.	Umunyinya	D	R, BR, and L	[42,43]
	*Albizia adianthifolia* (Schum.) W. Wight	Umusebeya/umusanza	D	L	[42]
Poaceae (2)	*Eragrostis racemosa* (Thunb.) Steud.	Ihumba	D	L	[49]
	*Sporobolus pyramidalis* P. Beauv.	Umutsina	Dysentery	S	[44]
Rosaceae (2)	*Prunus africana* (Hook. f.) Kalkm.	Umwumba	Dysentery	L	[33]
	*Rubus rigidus* Sm.	Umukeri	D	PWR	[44,46]
Ranunculaceae	*Clematis hirsuta* Guill. & Perr.	Umunkamba	D	L	[46,51]
Clusiaceae	*Harungana madagascariensis* Lam. ex Poir.	Umushayishayi	D	BS	[42,44]
Asparagaceae	*Dracaena afromontana* Mildb.	Umuhati	D	L	[44]
Moraceae	*Ficus thonningii* Blume	Umuvumu	Dysentery	L	[44]
Iridaceae	*Gladiolus psittacinus* Hook.	Karungu	D	R	[44]
Musaceae	*Musa acuminata* Colla	Igitoki cy’umushabi	C, dysentery	F	[44]
Myricaceae	*Myrica kandtiana* Engl.	Isubyo	D	BS and L	[44]
Pedaliaceae	*Sesamum angolense* Welw.	Igonde	Dysentery	L	[44,46]
Splanaceae	*Solanum dasyphyllum* Schum. et Thonn.	Igitoborwa	D	R and BR	[44]
Melastomataceae	*Dissotis throthae* Gilg	Icyeba	D	ND	[52]
Myrsinaceae	* Maesa lanceolate * Forssk.	Umuhanga	Dysentery	L	[48]
Olacaceae	* Ximenia caffra * Sond.	Umusasa	D	R	[53]
Annonaceae	*Annona senegalensis* Pers.	Umuhonnyo	D	S and BR	[54]
Melianthaceae	*Bersama abyssinica* Fresen	Umukaka	D	L	[55]
Caricaceae	*Carica papaya* L.	Ipapayi	DC	L	[43]
Lycopodiaceae	*Lycopodium clavatum* L.	Kazibannyo	D	WP	[43]
Plantaginaceae	*Plantago palmate* Hook.f.	Imbatabata	DD	R	[49]
Urticaceae	*Urtica massaica* Mildbr.	Igisura	D	L and S	[49]
Cucurbitaceae	*Momordica foetida* Schumach.	Umwishywa	D	L	[49]
Passifloraceae	*Passiflora edulis* Sims	Marakunja	D	L	[49]
Asphodelaceae	*Aloe spp.*	Igikakarubamba	D	L	[33]

Key: C, cholera; D, diarrhea; DD, diarrhea and dysentery; DC, diarrhea and cholera; DDC, diarrhea, dysentery, and cholera; L, leaves; F, fruits; S, stem; R, root; B, bark; H, herb; BR, bark root; BS, bark of stem; WP, whole plant; PWR, plant without root; and ND, not indicated. The number in brackets (first column) refers to the number of species belonging to each family. Table 1 is arranged in descending order according to the number of medicinal plants per family.

**Table 2:** Preparation and posology of some medicinal plants used for treating diarrheal cases.

Scientific Name	Local Name	Part Used	Preparation	References
*Vernonia amygdalina*	Umubilizi	L	Fresh leaves are ground, boiled in water with salt, and the resulting liquid is used	[42]
*Vernoniamiombicola* Willd	Idoma	L	Decoction of fresh leaves’ juice is used	[42,43]
*Guizotia scabra (Vis.) Chiov.*	Igishikashike	L	Fresh leaves are boiled in water and used to treat liver disease, intestinal worms, and diarrhea	[42]
*Phyllunthus bequaertii*	Uruheza	S and L	Extract of the stem and the leaves mixed with salt is used	[42]
*Tragia brevipes* (1), *Lagenaria sphaerica* (2), and *Euphorbia schimperiana* (3)	Isusa (1), umutanga (2), and kamaramahano (3)	L	To treat diarrhea, the extract of the crushed leaves of (1) is mixed with that of (2) and (3)	[43]
*Tetradenia riparia*	Umuravumba	L	Infusion or decoction is used	[56]
*Erythrina abyssinica*	Umuko	L	Leaves are boiled, and the obtained liquid is mixed with milk and taken while the solution is still hot	[43]
*Entada abyssinica*	Umunyinya	SB	Decoction of fresh stem bark is used	[42]
*Crotalaria mesopontica* (1), *Erythrococca bongensis* (2), and *Sida cordifolia* (3)	Akayogera (1)	L	Leaves of (1) are ground with the leaves of (2) and (3). Then, the mixture is boiled and filtered. A little salt is added to the filtrate	[43]
*Thunbergia alata*	Nkurimwonga	L	A glass of diluted extract is orally taken in the morning and another at noon	[43]
*Rumex usambarensis*	Umufumbegeshi	L	Fresh leaves are enclosed by banana leaves and put in charcoal for a while and the extracted juice is used	[43]
*Psidium guajava*	Ipera	L	Leaves are boiled and the resulting liquid is used	[43]
*Syzygium parvifolium*	Umugote	L	For this plant, currently known as Syzygium guineense, a decoction of leaves is used	[43]
*Mangifera indica* (1) and *Carica papaya* (2)	Umwembe (1) and ipapayi (2)	L	Leaves of (1) and those of (2) are ground and the obtained extract is mixed with water and then salt is added	[43]
*Chenopodium ugandae*	Umugombe	L	Leaves are ground and the extract is mixed with warm milk	[43]
*Chenopodium procerum*	Umwisheke	L	Leaves wrapped in a banana leaf are buried under the embers. Then, the aqueous solution is extracted	[43]
*Chenopodium* (1) *ugandae* and *Chenopodium procerum* (2)	Umugombe (1) and umwisheke (2)	L	Fresh leaves are crushed. The extract is filtrated and taken orally: two spoons three times/day (morning, midday, and evening) for 5 days	[49]
*Rubia cordifolia* L.	Umukararambwe	R	Roots are cleaned and ground in milk that has been kept fresh for a day, mixed with a little water, and the filtrate or a decoction is used	[42,43]
*Mitragyna rubrostipulata*	Umuziwaziwa/umuzibaziba	L	Leaves are boiled in water and the obtained solution is consumed	[42,43]
*Cyathula uncinulata*	Igifashi	L	Leaves are put on fire in a banana leaf and the juice is extracted	[43]
*Clematis hirsuta*	Umunkamba	L	Leaves are ground and extraction is done with water. To the filtrate, macerated sorghum flour is added	[43]
*Albizia adianthifoli*	Umusange/umusanza	L	Fresh leaves are ground, cooked in water with salt, and used to treat diarrhea	[42]
*Indigofera asparagoides*	Kizibana	H	A decoction of the crushed fresh herb is used	[42]
*Dracaena afromontana*	Umuhati	L	The leaves are first ground. Then, a little water and fresh milk (or porridge) are added. The mixture is allowed to macerate overnight	[43]
*Ficus thonningii* (1), *Mangifera indica* (2), and *Psidium guajava* (3)	Umuvumu (1)	L	The extract of (1) is mixed with the extracts of (2) and (3) in a little quantity of water	[43]
*Myrica kandtiana Engl.*	Isubyo	L	Leaves are ground with water and the extract is used	[43]
*Sporobolus pyramidalis*	Umutsina	S	The stem is ground and extraction is done with water	[43]
*Urtica massaica*	Igisura	L and S	To orally administer the macerated fresh leaves or the filtrate of dry and crushed stems: two spoons three times per day (morning, midday, and evening) for 5 days	[49]
*Plectranthus laxiflorus*	Umukuzanyana	L	To orally administer the filtrate of fresh leaves; one spoon three times per day (morning, midday, and evening) for 5 days	[49]
